# Prevalence of Dog Erythrocyte Antigen 1 in 7,414 Dogs in Italy

**DOI:** 10.1155/2017/5914629

**Published:** 2017-09-24

**Authors:** Anyela Andrea Medina Valentin, Alessandra Gavazza, George Lubas

**Affiliations:** Veterinary Transfusional Center (CTV), Department of Veterinary Sciences, University of Pisa, Via Livornese Lato Monte, San Piero a Grado, 56122 Pisa, Italy

## Abstract

The study aim was to establish the prevalence of DEA 1, the most immunogenic and clinically important blood group in canine blood transfusion, in 7,414 dogs from Italy. The potential sensitization risk following a first transfusion and the acute reaction risk following a second transfusion given without a cross-matching and blood typing test were also calculated. Dogs tested were purebred (4,798) and mongrel (2,616); 38.8% were DEA 1 negative and 61.2% were DEA 1 positive. High prevalence for DEA 1 positive blood type was found in Ariegeois and English Setter, whereas German Shepherd and Boxer had higher DEA 1 negative blood type. Breeds with blood type never reported before included French Brittany Spaniel and Pug showing a high prevalence of DEA 1 positive type, while French Bulldog and West Highland White Terrier were more often DEA 1 negative. Just 48.8% of purebred and 13.9% of mongrel dogs were considered as prospective blood donors based upon their blood type. Most of the breeds had a sensitization risk of 20.0–25.0%. Rottweiler and Ariegeois had less risk of sensitization (9.4 and 4.2%) and the minor risk of an acute transfusional reaction (0.9–0.2%). The prevalence of DEA 1 positive and negative dogs in Italy agrees with most of the data already reported in the literature.

## 1. Introduction

Canine blood groups are recognized and have standardized terminology as Dog Erythrocyte Antigen (DEA). The DEA system includes seven well-known blood groups (DEA 1, 3, 4, 5, 6, 7, and 8) with over twenty antigen specificities [1–4]. Other nonstandardized antigens within DEA such as Dal [3, 5] and the recently described Kai 1 and 2 have been reported [6]. Canine blood groups comprise a two-allele system with a positive and negative type.

The DEA 1 blood group system initially was described with 3 types, DEA 1.1, 1.2, and likely 1.3 [3, 7, 8]. Utilizing quantitative flow cytometry and an immunochromatographic technique with a monoclonal anti-DEA 1 alloantibody the continuum of DEA 1 negative to weakly (1+) up to strongly DEA 1 positive (4+) blood type was observed in contrast to the originally described DEA 1 system and with a significant correlation between these tests [9]. Recently, an autosomal dominant mode of inheritance of 4 alleles of DEA 1 with strong (4+) to weak (1+) reactivity was discovered. DEA 1 positive alleles are dominant over DEA 1 negative allele without any direct correlation with the historical DEA 1.2 positive subtype [10]. So now, it is accepted to speak about the DEA 1 group as a whole without any subtype [9, 10].

Natural occurring antibodies against DEA 1 antigen in dog's erythrocytes have never been positively identified [11]. On the other hand, natural antibodies against DEA 3, 5, and 7 have been documented with a prevalence of 6%, 23%, and 45%, respectively [2]. However, although all canine blood group antigens can stimulate the formation of alloantibodies, DEA 1 seems to be the most immunogenic and also is considered the most clinically important. Alloantibodies will appear following the first transfusion in DEA 1 negative recipient dogs receiving positive DEA 1 red blood cells (RBCs) within 4–14 days [11–14]. Sensitization of the recipient and production of alloantibodies can result in a severe acute hemolytic transfusion reaction and even death if a second DEA 1 positive RBC transfusion is administered to the same patient [15–17]. The risk of alloantibody production and transfusion reactions against antigens other than DEA 1 is not yet well defined [2] and there is no documented clinical evidence of a hemolytic reaction caused by DEA 1.2, 3, 5, and 7 in mismatched transfusions [18].

Blood typing to identify the presence of DEA 1 and the cross-match to establish full compatibility should be performed before each transfusion in order to reduce the risk of sensitization or immunological reaction between donor and recipient dogs [2, 19, 20]. Available methods for typing DEA 1.1 antigen include agglutination cards (RapidVet-H®, Agrolabo, Scarmagno, TO, Italy) and an immunochromatographic strip for DEA 1 (Quick Test DEA 1®, Alvedia, Lyon, France) both using monoclonal antibodies and are useful for in-clinic testing [17, 21]. A gel column agglutination test using microtubes is generally reserved for use in laboratory settings. The advantage of gel column typing is the ability to establish the degree of agglutination that can be graded from 0 to 4+. A DEA 1.1 positive result is considered if the reaction is graded as ≥2+ [17, 18, 22].

The knowledge of breed differences for prevalence of DEA 1 is very important for the recruitment of typed compatible blood donors. DEA 1 is expressed approximately in 40–60% of the general canine population. The prevalence of canine blood group DEA 1 has been studied in small populations in geographically restricted areas [23–28]. The most frequently studied breeds were German Shepherd, Golden Retriever, Greyhound, Doberman, and Rottweiler along with investigations in mongrel dogs [4, 18, 28–30].

The aim of our study was to determine the prevalence of blood type DEA 1 in a large population database of purebred and mongrel dogs reared in Italy. Furthermore, we calculated the potential risk of sensitization following a first transfusion and the subsequent risk of an acute transfusional reaction as documented immune-mediated hemolysis and/or agglutination following the second transfusion in the absence of a pretransfusional cross-match and blood typing test.

## 2. Material and Methods

The population of this study included 7,414 dogs retrieved from the Italian database of the website Dog blood Donors (DbD) (http://www.dogblooddonors.it). The data collected were recorded in the website during the period of October 2014–July 2016. The following information has been extracted from the DbD database: date of the dog's registration on the website, breed, weight, sex, year of birth, age, type of blood group (negative or positive DEA 1), and the Italian regions where the dog was living at the time of registration. Dogs were enrolled by private practitioners who recorded the above data into the DbD website.

DEA 1 blood type was established using two commercial in-clinic typing tests: RapidVet-H, Canine DEA 1.1, Agrolabo (Scarmagno, TO, Italy), and Quick TEST DEA 1 Alvedia (Lyon, France, distributed in Italy by Alcyon Italy, Marene, CN). Both tests use a monoclonal antibody, the former in an agglutination assay and the latter in immunochromatographic strips. Dogs were typed using one of the two in-clinic tests. Nevertheless, the RapidVet-H Canine DEA 1.1 test was the one most used in up to 95% of the dogs.

The data collected from the website DbD has been organized as follows:

- The prevalence of DEA 1 negative and positive types in the total population and in breeds that showed a substantial number of subjects registered (over 50) was calculated.
- The breeds numbering over 50 subjects was subtyped as females and males and the prevalence of DEA 1 was calculated according to sex.
- In the breeds numbering over 50 subjects the percentage of the breed's population was determined in relation to the total Italian canine population inferred from the last 10 years of registration at the ENCI (Ente Nazionale di Cinofilia Italiana, Italian National Kennel Club) (http://www.enci.it) which was calculated.
- The data concerning the provenience and corresponding blood type of the 7,414 dogs was subdivided into different geographical regions of Italy.
- Using the standard inclusion criteria for canine blood donors (dogs heavier than 25 kg and aged between 2 and 8 years) and breeds numbering over 20 subjects were considered as prospective “blood donors” regardless of whether they were DEA 1 positive or negative.
- The risk of sensitization for each breed (over 50 subjects) was calculated using the formula [(% DEA 1 negative ×  % DEA 1 positive)/100] to establish the probability of a dog to become sensitized after the first transfusion of blood without having been tested with a cross-match and typed for DEA 1 [28, 30, 31].
- Using the following formula [(% DEA 1 negative ×  % DEA 1 positive) × (% sensitization for the first transfusion/10,000)] the probability of each breed (over 50 subjects) to develop an acute hemolysis and/or agglutination (immune-mediated) transfusional reaction with a second incompatible transfusion using un-cross-matched and untyped blood was calculated [28, 30, 31].
- The Excel® 2016 software (Microsoft Office) was used for data analysis.

## 3. Results

In Table 1, the prevalence of DEA 1 negative and positive types in the canine population registered in the database of the DbD website in Italy (breeds represented by over 50 dogs) is reported. The prevalence of DEA 1 negative and positive types in breeds with less than 50 subjects and the complete list of breeds are not shown.

In Table 2, the prevalence of DEA 1 negative and positive types for females and males for breeds represented by over 50 dogs is reported.

In Table 3, the percentage of breed's population in relation to the total Italian canine population inferred from the last 10 years of registration at the ENCI for the breeds numbering over 50 subjects is reported.

In Table 4, the prevalence of prospective blood donors (PBD), using inclusion standard criteria for canine blood donors (including dogs heavier than 25 kgs and aged between 2 and 8 years) is reported. Only breeds with more than 20 subjects were considered.

In Table 5 the risk of sensitization following the first transfusion and the risk of an acute transfusional reaction documented as hemolysis and/or agglutination (immune-mediated) following the second transfusion in the absence of pretransfusional cross-match and blood typing test according to the most involved breeds were shown.

**Figure 1:**
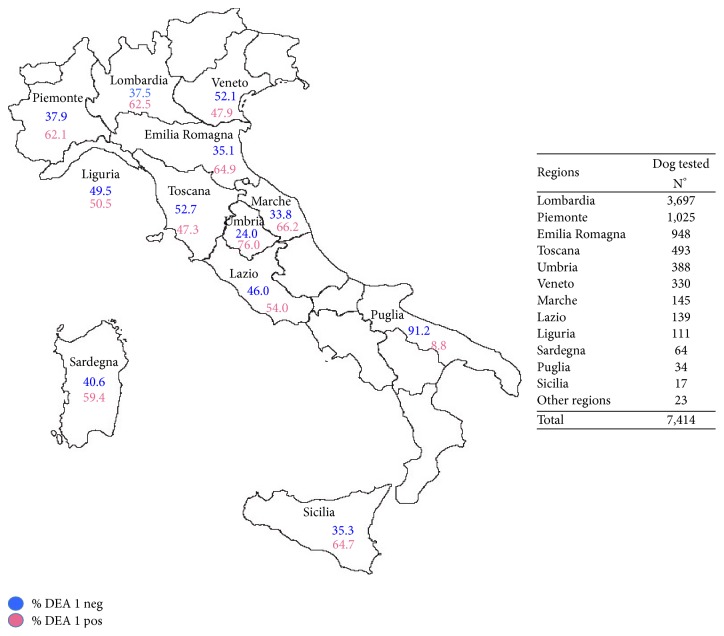
Distribution of DEA 1 negative and positive dogs in Italians regions (according to raw data retrieved from http://www.dogblooddonors.it website).

In Figure 1 the distribution of DEA 1 negative and positive types in the several Italians regions is shown.

## 4. Discussion

This paper reports the largest study among the references available on DEA 1 blood group prevalence to date. In addition, is also includes the DEA 1 prevalence in breeds never reported before. Indeed, a high number of purebred canine subjects have been tested here, and the results provide substantial information about the prevalence of DEA 1 blood group in Italy, which could be useful not only in transfusion medicine but also for canine genetic epidemiologic studies.

To obtain the most reliable results about the prevalence of DEA 1 in the canine purebred population in Italy, only breeds represented by over 50 dogs were analyzed. The prevalence of PBD in purebred and mixed dog's breeds represented by more than 20 subjects also was examined. Of total 7,414 dogs tested (purebreds 4,798, mongrel 2,616) 2,878 (38.8%) were DEA 1 negative and 4,536 (61.2%) were DEA 1 positive (Table 1). The prevalence for DEA 1 positive dogs found in this study was very similar to results 553/890 (62.0%) reported in another study carried out in Italy [29]. Furthermore, there have been other studies that showed similar results for DEA 1 positive dogs as in the case of Nigeria 71/178 (60.1%), Turkey 116/178 (65.2%), Portugal 156/274 (56.9%), and Spain 110/206 (53.4%) [4, 25, 28, 32]. Nevertheless, the first research about blood groups distribution in dogs from 1961 reported a lower prevalence of DEA 1 positive dogs 148/332 (44.6%) [13]. The difference here could reflect the effects of the ongoing worldwide inbreeding and linebreeding practiced within the more popular dog breeds over the ensuing years. A recent study in eastern Africa reported as much as 78/100 (78.0%) for DEA 1 positive dogs [33], representing the highest reported prevalence for DEA 1 positive blood types. Unfortunately, the paper did not report which breeds were included and the canine population studied was quite small. It is well known that the DEA 1 blood group distribution varies according to the geographical area investigated [6].

The DEA 1 blood group in dogs has been studied mainly for transfusional purposes. Transfusion reactions usually do not occur following the first transfusion because naturally occurring antibodies against DEA 1 are very rarely found in dogs (<0.3% in a population of 2,500 dogs tested by Hale and Werfelmann) [34], unless they have been previously sensitized by an incompatible transfusion. Regardless of this, DEA 1 negative dogs should receive only DEA 1 negative blood. If a negative DEA 1 dog is transfused with DEA 1 positive erythrocytes, an antibody reaction (agglutination and/or hemolysis) can be elicited in the recipient [2].

The good clinical practice in canine transfusion medicine requires that both recipient and donor should be blood typed for DEA 1, which is considered highly antigenic and the most clinically relevant blood type [2, 35]. In addition, the cross-match test is advisable to be carried out before a transfusion. If a recipient receives consecutives transfusions of incompatible blood, this patient could be highly sensitized and develop a severe acute hemolytic reaction [15, 16].

In this study a high prevalence for DEA 1 positive type in breeds such as Rottweiler (89.5%), Golden Retriever (75.2%), and Dachshund (74.2%) was reported. These results are comparable to those found in other previous investigations: 78.0–100% in Rottweiler, 77.0–95.0% in Golden Retriever, and 71.0–100% in Dachshund [24, 28, 29, 36, 37]. It is important to know the frequency of DEA 1 positive blood type in the Rottweiler and Golden Retriever as they could be enrolled as blood donors, because their weight is in the range established for donations and they are generally docile dogs. The high prevalence for breeds such as Ariegeois (95.7%), English Setter (81.0%), and Zwergpinscher (80.0%) has been confirmed according to the previous study carried out in Italy [29].

In this study, the Labrador Retriever breed showed a high prevalence for DEA 1 positive type (65.3%) as reported previously in studies carried out in Switzerland, South Africa, and Italy [24, 29, 37]. Nevertheless, in the published study from Portugal, Labrador Retrievers showed a higher prevalence for DEA 1 negative type (55.0%), although those results could be related to the low number of dogs (number 29) tested in comparison to our data (number 478) [28].

The prevalence of DEA 1 positive type in English Cocker Spaniel is 65.2%, in the Poodle 68.8%, and in Yorkshire Terrier 63.0%. These values are different from those found in other countries such as Switzerland which reported a high prevalence for DEA 1 negative (67%) type in the Poodle and in Portugal which reported 68.8% of DEA 1 negative type in the English Cocker Spaniel [28, 37].

In this study, the Shih Tzu breed showed a high prevalence for DEA 1 positive type (72.2%), while, on the contrary, a Japanese study reported an almost equal prevalence for DEA 1 positive (57%) and negative (43%) types in this breed. It should be pointed out, however, that only a small number of dogs (7) were tested in the Japanese study [38].

A high frequency for DEA 1 positive blood type has been observed in breeds such as French Brittany Spaniel (77.1%), Pug (77.0%), English Springer Spaniel (74.1%), and Chihuahua (61.0%). These results cannot be compared due to the absence of previous studies (Table 1).

In this study breeds with high DEA 1 negative prevalence were German Shepherd (81.1%), Boxer (83.0%), French Bulldog (77.9%), and West Highland White Terrier (63.0%) (Table 1). The high prevalence for DEA 1 negative type in German Shepherd was similar to that found previously in few countries such as South Africa (84.0%) [24], Portugal (100%) [28], Brazil, and Italy (both 90.0%) [29, 36]. Almost the same situation was observed in the Boxer, in addition to South Africa (88.0%) [24], Portugal (100%) [28], Switzerland (100%) [37], and Italy (80.0%) [29]. It is noteworthy that DEA 1 negative frequencies in French Bulldog (77.9%) and West Highland White Terrier (63.0%) were not previously reported. A high prevalence of DEA 1 negative type could be expected from the mating between female and male dogs within the same blood line but it is unknown if these tested dogs were the result of inbreeding or linebreeding. A recent study showed that mating between DEA 1 negative dogs strictly produced DEA 1 negative offspring, while mating between DEA 1 positive dogs primarily resulted in DEA 1 positive offspring, with an occasional DEA 1 negative offspring [10].

In our report, several breeds such as Jack Russell Terrier, Beagle, Border Collie, and Maltese showed an almost equal prevalence of DEA 1 negative and positive subjects. On the other hand, in this study, the Cane Corso breed shows exactly 50.0% for both DEA 1 negative and positive subjects, and these results are completely different from those obtained in another study carried out in Italy (72.0% for DEA negative type) [29]. The DEA 1 negative type prevalence reported in South Africa for American Staffordshire Terrier was 75.0% which differs from the equal prevalence found in the current study [24]. Another breed whose prevalence differs from those found in this study is the Maltese breed which reported a 33% for DEA 1 positive type in a study carried out on only 9 Maltese dogs in Japan [38]. The prevalence found in Cavalier King Charles Spaniel (48.5% of DEA 1 positive and 51.5% of DEA 1 negative types) dogs cannot be compared with any previous study as far as we know (Table 1).

Differences in the DEA 1 frequency in various countries are generally due to the selection of the population tested and the genetic drift deriving from local breeding. It is important to consider that in this study the sampling for DEA 1 testing was using dogs randomly accessed from several veterinary facilities and privately owned. However, as the blood type is a genetic marker, it should be considered as one way to reveal the hybrid history of canine breeds supplying information about migration and breed development in the canine database of several countries. Of course, this information is less accurate than the genomic data in the phylogenetic analysis reported by Parker et al. (2017) [39].

Breeds such as French Brittany Spaniel, Pug, English Springer Spaniel, and Chihuahua had a higher prevalence for DEA 1 positive type. On the other hand, breeds that had higher prevalence for DEA 1 negative type were French Bulldog and West Highland White Terrier. So far, there are no studies that report the DEA 1 prevalence for these breeds, although it would be interesting to be able to compare these results, even though we are not sure if the dogs included in this study from matings between inbred or tightly linebred dogs. Perhaps the fact that some of these dogs presented a high percentage of positive or negative DEA 1 blood type could be the result of matings between two DEA 1 negative dogs that produced only DEA 1 negative offspring or matings between DEA 1 positive dogs that resulted in DEA 1+ dogs, with an occasional production of a DEA 1 negative dog [10].

There was no notable difference in the prevalence for DEA 1 negative or positive type among females and males in this study, except for females Boxers (13/18 DEA 1 positive dogs were females), which represented 72.2%. The Cavalier King Charles Spaniel (35/50 DEA 1 positive dogs were males) and West Highland White Terrier (14/20 DEA 1 positive dogs were males) both represented 70%. The Ariegeois breed females had the highest DEA 1 negative (100%) type but only 4 females were tested (Table 2). It would be interesting to know if Ariegeois negative bitches had received blood transfusions so they could become sensitized. In this occurrence, the mating with a DEA 1 positive dog could raise the probability of risk of neonatal isoerythrolysis in any DEA 1 positive offspring [2].

There are not any studies about the prevalence of DEA 1 negative or positive types in relation to sex and breed except an investigation carried out in East Africa showing generic similar result but in a very small population [33]. A previous study established that the DEA 1 blood group system is an autosomal trait, with both male and female dogs either being DEA 1 negative or having varying degrees of DEA 1 positivity [10]. In addition, a survey ascertained that female dogs, even after pregnancy, did not develop any alloantibodies against RBC antigens during gestation, so they can be used safely as blood donors [40]. Further, additional pretransfusion compatibility testing is not required should they require transfusions themselves.

The obtained percentages of purebred dogs tested for DEA 1 in comparison to the numbers of these breeds enrolled by ENCI in the last 10 years were more than 1.0% in Yorkshire Terrier, Ariegeois, Shih Tzu, Maltese, and Cavalier King Charles Spaniel, and a high consistency (almost 3.0%) was noted in the Zwergpinscher breed (Table 3). These percentages provide important genetic epidemiologic data for the prevalence of DEA 1 in Italian purebred dogs.

The data compiled in the DbD website permitted us to achieve the aims of the project in the several Italian regions covered (Figure 1). The northern Italian regions were apparently more aware of the need for blood donation in dogs in comparison to the other Italian regions. This situation seems to be the same in humans blood donors where a higher percentage of donors were in regions such as Emilia Romagna (3.2%), Marche (3.5%), and Lombardia (2.6%) [41]. In addition, the low number of enrolled dogs in southern Italian regions could also be related to the low number of dogs officially registered with a microchip, since this data was a mandatory requirement to enroll dogs in the DbD website.

The so-called “universal donor” dogs are generally accepted as being negative for DEA 1, 3, 5, and 7 but positive only for DEA 4. Usually DEA 3, 5, and 7 blood groups are not tested because they do not show a major transfusion reaction during the first transfusion and there are difficulties in obtaining the required blood typing antisera [2].

Anyhow, the prevalence of alloantibodies against blood group antigens in dogs is quite rare and the only alloantibody consistently found is against DEA 7 antigen (around 10% in 2,500 dogs tested), and so far evidence of transfusion reactions or neonatal isoerythrolysis has not been documented [17, 34, 40].

In this study, 852/1745 (48.8%) of the purebred dogs were considered as PBD. A smaller number of PBD have been found in the mixed breed dogs (304/2616, 13.9%) (Table 4). The status of PBD was established using the standard inclusion criteria for canine blood donors as data reported in the DbD web page, namely, dogs being heavier than 25 kgs and of ages between 2 and 8 years, regardless of whether they were DEA 1 positive or negative and female or male (Table 4). Obviously, due to their selection as PBD dogs the distribution of DEA 1 negative and positive type is different from the general population studied and reported in Table 1.

Most of the breeds in this study have the sensitization risk ranging from 20.0 to 25.0% which means that the probability of a dog to become sensitized following the first transfusion of blood without having been tested with a cross-match and typed for DEA 1 is quite high (Table 5). Rottweiler and Ariegeois breeds had only a minor risk to become sensitized and produce antibodies against DEA 1 following the first transfusion (9.4 and 4.2%, resp.). These results are related to the higher prevalence of DEA 1 positive type in these breeds which reduced the risk of transfusional reactions. On the other hand, Beagle, Cane Corso, and Cavalier King Charles Spaniel had the highest percentage likelihood to become sensitized (25.0%) (Table 5).

Regarding the risk of an acute transfusional reaction documented as hemolysis and/or agglutination following the second transfusion, Ariegeois and Rottweiler breed showed the minor risk (0.2–0.9%). On the contrary Cane Corso, Beagle, Cavalier King Charles Spaniel, American Staffordshire Terrier, Border Collie, Jack Russell Terrier, and Maltese showed the highest risk (6.3 to 6.1%) (Table 5). Only a few studies have reported the risk for sensitization of an acute transfusional reaction. In 4 native breeds of Turkey a high sensitization risk after the first transfusion (14.4–35.6%) and the risk of an acute hemolytic reaction after second transfusion (7.2–25.3%) were found [32]. In Ibizan Hound and Galgos from Spain the sensitization risk after the first transfusion was similar to the data reported here [4, 30].

A limitation of this study was the use of the two in-clinic blood typing methods. Misclassification of blood typing results could occur even if performed by an experienced person [21]. Indeed, taking pictures of the results and storing them for future needs are suggested, but we were not able to have access to these raw data. Considering the high number of animals tested, however, even if a few errors in blood typing classification occurred, they would not have influenced the overall conclusions of the data presented. Finally, when used in healthy dogs both assays gave almost equivalent results [21].

Regardless of this, at present there is no a gold standard for blood typing in dogs [16]. The Quick TEST DEA 1 Alvedia and the RapidVet-H Canine DEA 1.1 are easy to use and were performed by veterinary practitioners. Nevertheless, the proper use and interpretation of the rapid typing test RapidVet-H Canine DEA 1.1, which was largely used in this study, were completed upon request by the Agrolabo, TO, Italy technical support group [8, 21, 22, 42].

## 5. Conclusions

This study provides an overview about the distribution of DEA 1 blood group in a large dog population belonging to purebred and mongrel dogs reared in Italy. The data could be useful in clinical transfusion medicine and for studies of canine genetic epidemiology. The prevalence of DEA 1 positive and negative dogs mostly agrees with previous prevalence studies reported in the literature. In addition, DEA 1 distribution was studied in breeds never previously reported. The risk of sensitization following the first transfusion and the risk of an acute transfusional reaction documented as hemolysis and/or agglutination following the second transfusion in the absence of a pretransfusional cross-match and blood typing test also was determined in most of the breeds studied.

## Figures and Tables

**Table 1:** Prevalence of DEA 1 negative and positive in the breeds.

Breeds	Dogs tested	DEA 1 neg	DEA 1 pos
	No.	No. (%)	No. (%)
Mixed	2,616	924 (35.3)	1,692 (64.7)
Labrador Retriever	478	166 (34.7)	312 (65.3)
German Shepherd	312	253 (81.1)	59 (18.9)
Golden Retriever	214	53 (24.8)	161 (75.2)
Jack Russell Terrier	213	100 (46.9)	113 (53.1)
Zwergpinscher	194	62 (32.0)	132 (68.0)
Chihuahua	164	64 (39.0)	100 (61.0)
English Cocker Spaniel	158	55 (34.8)	103 (65.2)
American Staffordshire Terrier	153	72 (53.1)	81 (46.9)
Dachshund	149	35 (25.8)	114 (74.2)
Poodle	144	45 (31.3)	99 (68.8)
Maltese	130	59 (45.4)	71 (54.6)
English Setter	126	24 (19.0)	102 (81.0)
Border Collie	124	66 (53.2)	58 (46.8)
Beagle	123	61 (49.6)	62 (50.4)
Yorkshire Terrier	108	40 (37.0)	68 (63.0)
Boxer	106	88 (83.0)	18 (17.0)
Cavalier King Charles Spaniel	103	53 (51.5)	50 (48.5)
Ariegeois	92	4 (4.3)	88 (95.7)
French Bulldog	86	67 (77.9)	19 (22.1)
French Brittany Spaniel	83	19 (22.9)	64 (77.1)
Shih Tzu	79	22 (27.8)	57 (72.2)
Cane Corso	76	38 (50.0)	38 (50.0)
Pug	61	14 (23.0)	47 (77.0)
Rottweiler	57	6 (10.5)	51 (89.5)
English Springer Spaniel	54	14 (25.9)	40 (74.1)
West Highland White Terrier	54	34 (63.0)	20 (37.0)
Other^*∗*^	1,159	440 (38.0)	719 (62.0)

*Total*	7,414	2,878 (38.8)	4,536 (61.2)

^*∗*^Breed represented by less than 50 dogs. neg, negative, pos, positive, No., total number, and %, percentage (according to raw data retrieved from http://www.dogblooddonors.it website).

**Table 2:** Prevalence of DEA 1 negative and positive in female and male dogs.

Breeds	Dogs tested	DEA 1 neg		DEA 1 pos	
		Females	Males	Females	Males
	No.	No. (%)	No. (%)	No. (%)	No. (%)
Mixed	2,616	475 (51.4)	449 (48.6)	895 (52.9)	797 (47.1)
Labrador Retriever	478	80 (48.2)	86 (51.8)	160 (51.3)	152 (48.7)
German Shepherd	312	120 (47.4)	133 (52.6)	25 (42.4)	34 (57.6)
Golden Retriever	214	33 (62.3)	20 (37.7)	85 (52.8)	76 (47.2)
Jack Russell Terrier	213	4 8 (41.0)	59 (59.0)	54 (47.8)	59 (52.2)
Zwergpinscher	194	28 (45.2)	34 (54.8)	66 (50.0)	66 (50.0)
Chihuahua	164	32 (50.0)	32 (50.0)	46 (46.0)	54 (54.0)
English Cocker Spaniel	158	28 (50.9)	27 (49.1)	52 (50.5)	51 (49.5)
American Staffordshire Terrier	153	37 (51.4)	35 (48.6)	37 (45.7)	44 (54.3)
Dachshund	149	21 (60.0)	14 (40.0)	58 (50.9)	56 (49.1)
Poodle	144	22(48.9)	23 (51.1)	40 (40.4)	59 (59.6)
Maltese	130	33 (55.9)	26 (44.1)	39 (54.9)	32 (45.1)
English Setter	126	12 (50.0)	12 (50.0)	38 (37.3)	64 (62.7)
Border Collie	124	37 (56.1)	29 (43.9)	32 (55.2)	26 (44.8)
Beagle	123	30 (49.2)	31 (50.8)	25 (40.3)	37 (59.7)
Yorkshire Terrier	108	20 (50.0)	20 (50.0)	36 (52.9)	32 (47.1)
Boxer	106	40 (45.5)	48 (54.5)	13 (72.2)	5 (27.8)
Cavalier King Charles Spaniel	103	20 (37.7)	33 (62.3)	15 (30.0)	35 (70.0)
Ariegeois	92	4 (100.0)	0 (0.0)	42 (47.7)	46 (52.3)
French Bulldog	86	29 (43.3)	38 (56.7)	8 (42.1)	11 (57.9)
French Brittany Spaniel	83	13 (68.4)	6 (31.6)	26 (41.9)	38 (61.3)
Shih Tzu	79	8 (36.4)	14 (63.6)	22 (38.6)	35 (61.4)
Cane Corso	76	15 (39.5)	23 (60.5)	22 (57.9)	16 (42.1)
Pug	61	6 (42.9)	8 (57.1)	19 (40.4)	28 (59.6)
Rottweiler	57	3 (50.0)	3 (50.0)	28 (54.9)	23 (45.1)
English Springer Spaniel	54	7 (50.0)	7 (50.0)	17 (42.5)	23 (57.5)
West Highland White Terrier	54	16 (47.1)	18 (52.9)	6 (30.0)	14 (70.0)
Other^*∗*^	1,159	219 (49.8)	221 (50.2)	353 (49.1)	366 (50.9)

*Total*	7,414	1,429 (19.3)	1,449 (19.5)	2,259 (30.5)	2,277 (30.7)

^*∗*^Breed represented by less than 50 dogs; neg, negative, pos, positive, No., total number, %, percentage (according to raw data retrieved from http://www.dogblooddonors.it web site).

**Table 3:** Breeds in relation to the overall size of dog breed population according to the ENCI last 10 years of registration.

Breeds	No. of dogs tested	ENCI data	%
Zwergpinscher	194	6,732	2.88
Yorkshire Terrier	108	7,664	1.41
Ariegeois	92	7,174	1.28
Shih Tzu	79	6,620	1.19
Maltese	130	12,816	1.01
Cavalier King Charles Spaniel	103	10,291	1.00
Poodle	144	15,185	0.95
French Bulldog	86	9,551	0.90
English Cocker Spaniel	158	20,528	0.77
West Highland White Terrier	54	7,297	0.74
Beagle	123	17,407	0.71
Pug	61	9,099	0.67
American Staffordshire Terrier	153	23,011	0.66
Labrador Retriever	478	77,116	0.62
Border Collie	124	23,101	0.54
Dachshund	149	28,108	0.53
Chihuahua	164	32,494	0.50
Jack Russell Terrier	213	46,221	0.46
Golden Retriever	214	49,580	0.43
Boxer	106	36,926	0.29
English Springer Spaniel	54	20,473	0.26
Cane Corso	76	31,296	0.24
German Shepherd	312	152,649	0.20
Rottweiler	57	30,350	0.19
French Brittany Spaniel	83	44,534	0.19
English Setter	126	145,906	0.09

ENCI: Italian National Kennel Club, No.: total number, %, percentage (according to raw data retrieved from http://www.dogblooddonors.it website).

**Table 4:** Number of prospective blood donors.

Breeds		DEA 1 neg	DEA 1 pos	^*∗*^PBD	DEA neg	DEA pos
	No.	No. (%)	No. (%)	No.	No. (%)	No. (%)
Labrador Retriever	478	166 (34.7)	312 (65.3)	264	105 (39.8)	159 (60.2)
German Shepherd	312	253 (81.1)	59 (18.9)	171	145 (84.8)	26 (15.2)
Golden Retriever	214	53 (24.8)	161 (75.2)	133	38 (28.6)	95 (71.4)
American Staffordshire Terrier	153	72 (53.1)	81 (46.9)	58	24 (41.4)	34 (58.6)
Boxer	106	88 (83.0)	18 (17.0)	66	56 (84.8)	10 (15.2)
Ariegeois	92	4 (4.3)	88 (95.7)	5	1 (20.0)	4 (80.0)
Cane Corso	76	38 (50.0)	38 (50.0)	27	17 (63.0)	10 (37.0)
Rottweiler	57	6 (10.5)	51 (89.5)	32	3 (9.4)	29 (90.6)
Bernese Mountain Dog	44	8 (18.2)	36 (81.8)	33	7 (21.2)	26 (78.8)
Dobermann	32	25 (78.1)	7 (21.9)	19	14 (73.7)	5 (26.3)
Kurzhaar	30	7 (23.3)	23 (76.7)	4	2 (50.0)	2 (50.0)
Australian Shepherd	29	9 (31.0)	20 (69.0)	3	0 (0.0)	3 (100.0)
Siberian Husky	29	12 (41.4)	17 58.6)	4	2 (50.0)	2 (50.0)
Maremma Sheepdog	24	12 (50.0)	12 (50.0)	15	7 (46.7)	8 (53.3)
Weimaraner	24	14 (58.3)	10 (41.7)	13	7 (53.8)	6 (46.2)
Belgian Shepherd	23	12 (52.2)	11 (47.8)	3	2 (66.7)	1 (33.3)
English Pointer	22	8 (36.4)	14 (63.6)	2	1 (50.0)	1 (50.0)

*Total*	1,745	787 (45.1)	958 (54.9)	852	431 (50.6)	421 (49.4)

^*∗*^PBD prospective blood donor based on age (2 to 8 years old) and body weight (>25 kg); neg, negative, pos, positive, No., total number, and %, percentage (according to raw data retrieved from http://www.dogblooddonors.it website).

**Table 5:** The risk of sensitization after 1st transfusion and the risk of an acute hemolytic reaction after 2nd transfusion in the absence of pretransfusional cross-match and blood typing test^§^.

Breeds	Sensitization risk after 1st transfusion (%)	Acute immune-mediated reaction after 2nd transfusion (%)
Beagle	25.0	6.2
Cane Corso	25.0	6.3
Cavalier King Charles Spaniel	25.0	6.2
American Staffordshire Terrier	24.9	6.2
Border Collie	24.9	6.2
Jack Russell Terrier	24.9	6.2
Maltese	24.8	6.1
Chihuahua	23.8	5.7
West Highland White Terrier	23.3	5.4
Yorkshire Terrier	23.3	5.4
Mixed	22.8	5.2
English Cocker Spaniel	22.7	5.1
Labrador Retriever	22.7	5.1
Zwergpinscher	21.7	4.7
Poodle	21.5	4.6
Shih Tzu	20.1	4.0
English Springer Spaniel	19.2	3.7
Dachshund	19.1	3.7
Golden Retriever	18.6	3.5
French Brittany Spaniel	17.7	3.1
Pug	17.7	3.1
French Bulldog	17.2	3.0
English Setter	15.4	2.4
German Shepherd	15.3	2.4
Boxer	14.1	2.0
Rottweiler	9.4	0.9
Ariegeois	4.2	0.2

^§^According to raw data retrieved from http://www.dogblooddonors.it website.
